# MHC-I alleles mediate clearance and antibody response to the zoonotic Lassa virus in *Mastomys* rodent reservoirs

**DOI:** 10.1371/journal.pntd.0011984

**Published:** 2024-02-29

**Authors:** Ayodeji Olayemi, Dominik Werner Schmid, Ramona Fleischer, Kerstin Wilhelm, Alexander Christoph Heni, Nadine Mueller-Klein, Lavinia Haikukutu, Elisabeth Fichet-Calvet, Stephan Günther, Simone Sommer

**Affiliations:** 1 Natural History Museum, Obafemi Awolowo University, Ile Ife, Osun State, Nigeria; 2 Institute of Evolutionary Ecology and Conservation Genomics, University of Ulm, Ulm, Germany; 3 Department of Wildlife Management, Sokoine University of Agriculture, Morogoro, Tanzania; 4 Department of Zoonoses Control, Bernhard Nocht Institute for Tropical Medicine, Hamburg, Germany; 5 Department of Virology, Bernhard Nocht Institute for Tropical Medicine, Hamburg, Germany; Seoul National University College of Medicine, REPUBLIC OF KOREA

## Abstract

West African *Mastomys* rodents are the primary reservoir of the zoonotic Lassa virus (LASV). The virus causes haemorrhagic Lassa fever and considerable mortality in humans. To date, the role of *Mastomys* immunogenetics in resistance to, and persistence of, LASV infections is largely unknown. Here, we investigated the role of Major Histocompatibility Complex class I (MHC-I) on LASV infection status (i.e., active vs. cleared infection, determined via PCR and an immunofluorescence assay on IgG antibodies, respectively) in *Mastomys natalensis* and *M*. *erythroleucus* sampled within southwestern Nigeria. We identified more than 190 and 90 MHC-I alleles by Illumina high throughput-sequencing in *M*. *natalensis* and *M*. *erythroleucus*, respectively, with different MHC allele compositions and frequencies between LASV endemic and non-endemic sites. In *M*. *natalensis*, the MHC allele ManaMHC-I*006 was negatively associated with active infections (PCR-positive) and positively associated with cleared infections (IgG-positive) simultaneously, suggesting efficient immune responses that facilitate LASV clearance in animals carrying this allele. Contrarily, alleles ManaMHC-I*008 and ManaMHC-I*021 in *M*. *natalensis*, and MaerMHC-I*008 in *M*. *erythroleucus*, were positively associated with active infection, implying susceptibility. Alleles associated with susceptibility shared a glutamic acid at the positively selected codon 57, while ManaMHC-I*006 featured an arginine. There was no link between number of MHC alleles per *Mastomys* individual and LASV prevalence. Thus, specific alleles, but not MHC diversity *per se*, seem to mediate antibody responses to viremia. We conclude that co-evolution with LASV likely shaped the MHC-I diversity of the main LASV reservoirs in southwestern Nigeria, and that information on reservoir immunogenetics may hold insights into transmission dynamics and zoonotic spillover risks.

## Author summary

The Lassa virus is passed from rodents to humans, causing a disease that kills thousands of people every year in West Africa. To control spread, scientists are trying to understand why some rodents are more susceptible to the virus than others. One factor they are studying is the genetic makeup of the rodents’ immune systems. They found that a specific allele of the Major Histocompatibility Complex (MHC I), called ManaMHC-I*006, was associated with resistance to and clearance of the virus, while other alleles were associated with susceptibility. They also found that the immunogenetic composition of rodents differed between areas where Lassa virus is endemic or non-endemic. These findings can help predict where the virus might emerge and can help researchers develop a vaccine to protect the rodents from getting the virus, which can ultimately help prevent the spread of the disease to humans. The authors emphasize the significance of adopting a One-Health approach in thinking about wildlife and human health.

## 1. Introduction

Lassa fever, caused by the Lassa virus (LASV), is a lethal zoonotic haemorrhagic disease endemic to West Africa, where it is estimated to cause up to 18,000 human deaths annually [1]. Infection during pregnancy has been reported to result in up to 50% maternal- and 87% foetal mortality [2–4]. About 30% of survivors from this illness suffer sensorineural hearing loss [5]. With a rising number of cases (Nigeria accounting for up to 90–99% of all clinical diagnoses [6]), the possibility of Lassa fever spreading outside the West African region is cause for concern [7,8]. Unsurprisingly, in the recently revised WHO R&D blueprint on emerging infectious diseases, LASV control was marked as high priority [9–11].

The main natural reservoir for LASV are rodents of the family Muridae [12], which do not display significant adverse effects from infection [13]. Humans can contract LASV when they come into contact with excreta of viruric rodents [14]. LASV infections in humans are generally congruent with distribution of the virus across rodent populations. However, this virus distribution tends to be concentrated in particular hotspots [15]. Projecting which rodent individuals and populations are prone to LASV occurrence will help predict the likelihood of emergence and, accordingly, transmission to humans.

The Natal multimammate mouse *Mastomys natalensis*, the most widespread and abundant rodent across Africa [16], is recognized as the primary LASV reservoir [12]. It is the natural host of a range of additional mammarenaviruses in and beyond West Africa (e.g., Dhati Welel [17], Gairo [18], Luna [19], Mobala-like [20], Mopeia [21] and Morogoro [22] viruses), and also host to other zoonotic pathogens (e.g., *Yersinia pestis* [23], *Leptospira* sp. [24], and *Trypanosoma* sp. [25]). Aside from *M*. *natalensis*, the Guinea multimammate mouse *M*. *erythroleucus* and other murid rodent species have been demonstrated as independent LASV reservoirs [26]. Though similar in external morphology and ecology, *M*. *natalensis* and *M*. *erythroleucus* are distinct species possessing different chromosome numbers (2n = 32 and 2n = 38, respectively [27]). Geographically, *M*. *erythroleucus* is limited to West- and Central Africa, where it lives in sympatry with the more widely distributed *M*. *natalensis* [27]. Yet, there are locations where only one species is present within Nigeria. Even though these multimammate mice function as reservoirs of human LASV, details concerning ecology of the virus in rodents and its connection to host immunogenetics are still unclear.

The Major Histocompatibility Complex (MHC) is a group of genes that mediate the recognition and presentation of self and non-self peptides on cell surfaces in order to trigger adaptive immune responses [28,29]. Glycoproteins coded by MHC-I primarily bind to peptides from intracellular microbes (e.g., viruses), whereas those coded by MHC-II predominantly bind to peptides from extracellular organisms (e.g., bacteria and helminths) [30–32]. MHC-II composition and diversity have been associated with resistance to parasites in a variety of non-model animal hosts [33–35] including wild Muridae (e.g., [36–38]). The allelic diversity at the MHC-II region of *Mastomys natalensis* was described previously [39]; while, for MHC-I, only orthologous sequences of the *Mastomys coucha* genome were identified [40]. Generally, descriptions of MHC-I composition and diversity in wild Muridae are scant. *Mastomys* MHC-I has never been investigated or linked to mammarenaviruses like LASV, even though MHC-I-mediated clearance of particular LASV peptides was already found more than a decade ago [41]. This is surprising given the zoonotic potential of this rodent family that copes well with habitat changes and often lives in close proximity to humans [42,43].

To fill this knowledge gap, we aimed to decipher the allelic MHC-I diversity in two *Mastomys* species (*M*. *natalensis* and *M*. *erythroleucus*) captured from two sites within southwestern Nigeria—one where LASV is endemic and the other with no history of infected rodents–in order to identify links between MHC-I genotype and LASV infection. Apart from rodents infected as neonates (which develop a perpetual infection, perhaps for life), the majority of LASV (and other closely-related mammarenavirus) infections in *Mastomys* are acute and transient, with individuals retaining anti-LASV IgG antibodies after virus clearance [44–47]. Consequently, individuals can be classified as carrying an ongoing LASV infection if PCR-positive or having cleared a past infection if IgG-positive. Regarding active infections, a recent capture-recapture study demonstrated that, even among individuals generally considered to be acutely infected, some carry the virus longer than others [48]. Lingering infections promote rodent-to-rodent transmission, and eventual spillover to humans. But, whether individual MHC-I composition is linked to the capacity to efficiently clear LASV remains an open question.

To investigate the link between immunogenetics and LASV infection, we used a co-occurrence analysis approach, combining *Mastomys* LASV infection status assessment with high throughput sequencing of MHC-I exon 2 to evaluate whether certain alleles were significantly a) negatively associated with active LASV infections (indicating resistance), b) positively associated with active infections (indicating susceptibility), and c) positively associated with cleared infections (indicating capability to mount an effective antibody response). Since we do not have any information on infection intensity or sero-levels of IgG [49], we define resistance and susceptibility as a rodent’s ability to effectively and timely clear an active LASV infection. We discuss our results in light of site-specific differences in LASV endemism since reservoir immunogenetics may provide insights into transmission dynamics and zoonotic spillover risks.

## 2. Methods

### 2.1 Ethics statement

This study analysed tissues obtained from *Mastomys* rodents trapped as part of our larger project, “Seroprevalence, Incidence and Risk Factors of Lassa Fever, Edo State, Nigeria”, approved by the Health Research Ethics Committee of the Irrua Specialist Teaching Hospital in Edo State, Nigeria (ISTH/HREC/2017/1019/28). No animal experiments were conducted. Rodents were euthanised using the anaesthetic isoflurane before biopsy collection, with strict safety precautions observed during their capture and necropsy [50].

### 2.2 Study area and sample collection

**Fig 1:**
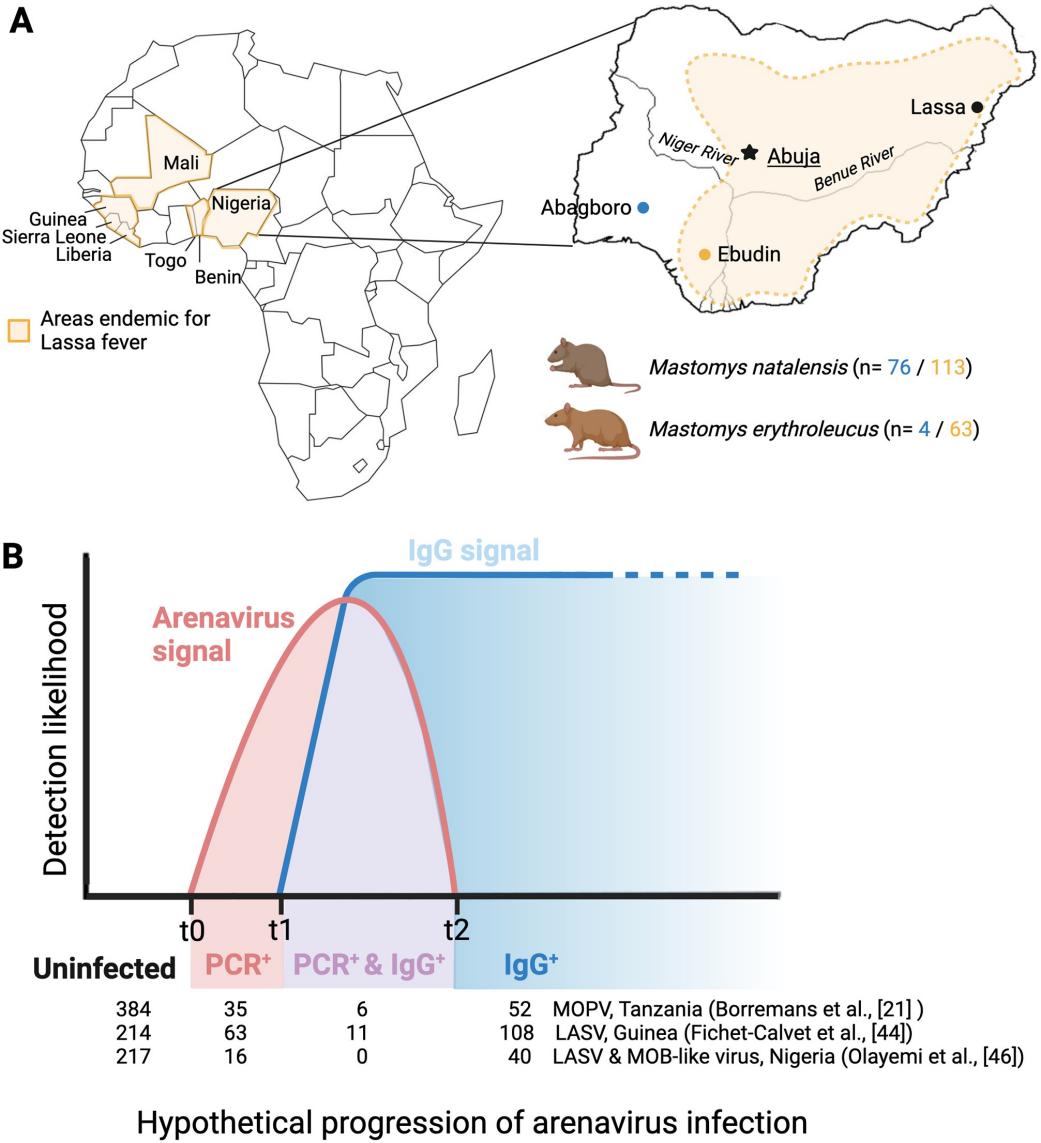
Geographic distribution of Lassa fever, depiction of the study area and the acute nature of most mammarenavirus infections in *Mastomys* rodents. A) Countries endemic for Lassa fever across West Africa, and endemic (Ebudin in yellow) and non-endemic (Abagboro in blue) sampling locations for *Mastomys* rodents within Nigeria. Sample sizes for *M*. *natalensis* and *M*. *erythroleucus* analysed for MHC-I in Abagboro and Ebudin are indicated below the Nigerian map. B) Schematic representation of the course of an acute infection with LASV and other closely-related mammarenaviruses hosted by natural populations of the rodent *Mastomys natalensis*. Infection progresses from active infection detected only via PCR (PCR^+^, t0 –t1), active infection and initiated seroconversion (PCR^+^ & IgG^+^, t1- t2), to IgG antibody presence without active infection (IgG^+^, > t2), indicative of a cleared infection. The table below the graph summarises the results from various mammarenavirus surveys, and how many rodents were identified to be acutely infected without showing an IgG response, infected and showing IgG responses, and only showing an IgG signal. Note: individuals found within the t1 –t2 category are in the middle of seroconversion; or denote chronic infections (not represented in the graph) in rodents infected from when they were newly born. MOPV: Mopeia virus. MOB-like: Mobala-like virus. The figure was created with BioRender.com.

The geographic distribution of Lassa fever in Africa, the location of the study sites within Nigeria and the schematic representation of the course of an acute infection with LASV and other closely-related mammarenaviruses hosted by natural populations of the rodent *Mastomys natalensis* is detailed in Fig 1. *Mastomys* were captured during previous ecological investigations to screen for LASV in southwestern Nigeria [20,51], and comprised 204 specimens of *Mastomys natalensis* and 69 of *M*. *erythroleucus*. Both *Mastomys* species were captured in Abagboro (7° 32´N, 4° 30´E) during January and September of 2011 and 2012 and in Ebudin (6° 35´N, 6° 10´E, approximately 292 km apart) during July, October, January and April from 2014 to 2016. Ebudin is located in the endemic zone for Lassa fever in Nigeria, while Abagboro is considered to be in the non-epidemic part of the country. Rodent sampling in Ebudin, where LASV is frequently detected in *Mastomys* rodents [51], allowed us explore the association between the occurrence of MHC-I alleles and LASV prevalence. Additionally, trapping in Abagboro enabled us to compare MHC-I constitution in rodents between endemic and non-endemic localities.

Rodents were captured in houses and surrounding vegetation using Sherman live-traps. Specimens were euthanised with the anaesthetic isoflurane and blood drawn by cardiac puncture. Using whole-blood samples, active LASV infection was determined via PCR [20,46,51] and previous infection by an Immunofluorescence Assay (IFA) to detect IgG antibodies [46,52]. The virus strains from all PCR-positive *Mastomys* individuals in this study belong to LASV lineage II [51], known to circulate in southern Nigeria. Because *Mastomys* species can be difficult to distinguish by external morphology, individual rodents were identified by sequencing a segment of mitochondrial Cytochrome *b* DNA from ethanol-preserved rodent kidneys, extracted using the DNeasy Blood & Tissue kit (Qiagen, Germany) according to manufacturer’s instructions. Genomic DNA extracted from rodent kidneys was also used for MHC-I characterization.

### 2.3 Primer design and library preparation

To the best of our knowledge, MHC-I has not been sequenced previously for rodents of the genus *Mastomys*. Aligning the few publicly available MHC-I sequences from the rodent family Muridae (e.g., [40,53]) in Geneious Prime version 2021.2 (Biomatters Inc., USA), we designed primers to amplify MHC-I exon 2 of *Mastomys* specimens using Primer3 [54]. The primer sequences Ma16F (forward primer, YCCCAGGCWCACACTCRMTGCG) and Ma257R (reverse primer, CTCACCGYCCTCGCTCTGG) are located mainly within the 270 bp of exon 2, with only 6 and 5 bp, respectively, extending into adjacent introns.

In order to generate an Illumina MiSeq DNA Amplicon library, a two-step PCR was carried out following the four-primer amplicon tagging scheme of Fluidigm (Access Array System for Illumina Sequencing Systems, Fluidigm, USA). For the first target specific (TS) PCR the primers were appended with either a CS1 adapter (CS1-4N-Ma16F) or CS2 adapter (CS2-Ma257). Four random bases (4N) were added to the forward primer to facilitate cluster identification on the Illumina flow cell. During the second barcoding PCR a sample-specific barcode (10bp) and Illumina adapter sequences were added to the CS adapters. The first TS-PCR of 10 μl-units included 1 μl template DNA, 5 μl AmpliGold 360 MasterMix (Thermo Fisher Scientific, USA), 1 μl GC buffer, 300nM of each CS primer and 2.4 μl ultrapure water. The temperature profile consisted of an initial denaturation phase at 95°C for 600 s; 30 cycles of denaturation at 95°C for 30 s, annealing at 59°C for 30 s, elongation at 72°C for 45 s; and a final elongation at 72°C for 420 s. Our MHC-I fragment targeted by PCR (240 bp) was examined for strength of amplification and purity on a QIAxcel Advanced System (Qiagen, Germany).

The second 20 μl PCR contained 2 μl TS-PCR product, 10 μl AmpliGold 360 MasterMix, 4 μl unique Fluidigm barcode primers, and 4 μl ultrapure water. Apart from a reduction to 10 amplification cycles and annealing at 60°C, the PCR protocol was equal to the first PCR. Distinct barcodes were also assigned to samples serving as replicates (*M*. *natalensis*: 28 out of 204 individuals; *M*. *erythroleucus*: 8 out of 69 individuals) (S2 Appendix). The barcoded PCR product was cleaned by using magnetic beads (NucleoMag NGS Clean-up and Size Select, Macherey-Nagel, Germany) and quality checked on the QIAxcel Advanced System. Concentrations for each amplicon were determined with QuantiFlour dsDNA System (Promega, USA) on a microplate reader (Tecan, Switzerland), and equimolar DNA amounts were pooled for the final library. A paired-end sequencing run was performed on an Illumina Miseq using a V2 reagent kit (2x250 cycles).

### 2.4 MHC allele calling

Our Illumina run produced 8,299,036 raw reads for *M*. *natalensis* and 3,082,197 for *M*. *erythroleucus* (S1 Appendix). Sequences were analyzed using the “Allele Calling Procedure for Illumina Amplicon Sequencing” pipeline (ACACIA; [55,56]) running both species separately. Essentially, ACACIA designates alleles from raw reads while removing sequences that represent chimeras, reading errors or singletons. In brief, we used the default settings provided by the pipeline for quality control, forward and reverse read merging, primer filtering and discarding of artifacts (singletons and chimeras). The remaining sequences were blasted against a reference database consisting of 754 MHC-I sequences (principally exon 2, with some sequences including exon 3) from 16 murid species extracted from NCBI (www.ncbi.nih.gov), resulting in 3,986,903 reads for *M*. *natalensis* and 1,465,859 for *M*. *erythroleucus*. For allele calling, we increased the entropy level from 0.2 to 0.35 to deal with extremely high genetic diversity and set the minimum number of reads for retaining a sequence as an MHC allele in an individual to 20. We reached a replicability of 93.83% and 99.12% for the 28 and 8 replicate samples from *M*. *natalensis* and *M*. *erythroleucus*, respectively.

Based on the ACACIA results, and in keeping with our conservative approach to oligotyping, we excluded nine individuals of *M*. *natalensis* and two of *M*. *erythroleucus* with less than 15,000 raw reads generated in the Illumina sequencing. We removed an additional five *M*. *natalensis*, which we considered to have an abnormally low number of alleles per individual (2–8), in light of the extent of MHC-I loci detected for this species (up to 22). Ultimately, 189 individuals of *M*. *natalensis* and 67 *M*. *erythroleucus* were retained for subsequent analyses (S1 Appendix). Details such as the nucleotide sequence and frequency of each MHC-I allele are contained in S2 Appendix.

### 2.5 MHC-I supertyping

Apart from individual alleles, we sought to investigate whether LASV prevalence was linked to the occurrence of MHC-I supertypes (STs), i.e., alleles grouped according to pathogen-recognition properties [57,58]. Identification of positive selection across codon sites in this study was conducted separately for *M*. *erythroleucus* and *M*. *natalensis* using CodeML in the PamlX graphical user interface [59]. The analysis detected 16 positively selected sites (PSSs), 12 of which are common to both species (S3 Appendix). MHC-I alleles were then clustered into supertypes (STs) based on five z-values [60] that described the physicochemical binding properties of each amino acid at the 12 shared PSSs using DAPC clustering (DAPC, [61]). Hence, MHC supertypes summarize alleles with putatively similar antigen binding characteristics. The optimal number of STs for our z matrix was suggested by the Bayesian Information Criterion (BIC) curve (S4 Appendix). We retained only alleles that were convincingly assigned (i.e., in at least 7 out of 10 DAPC seed runs) within a particular ST. This resulted in 225 *M*. *erythroleucus* and *M*. *natalensis* alleles allocated to 24 distinct supertypes (S4 Appendix).

### 2.6 Statistical analyses

MHC allele and supertype composition between *Mastomys* species (*M*. *natalensis* and *M*. *erythroleucus*) and between localities (Abagboro and Ebudin) were compared using a PERMANOVA computed with the *adonis()* function in the ‘vegan’ package [62, 63]. Significant association between the occurrence of individual MHC alleles, supertypes and active or previous LASV infection was assessed with the *cooccur()* function from the ‘cooccur’ package [64]. Only alleles and STs present in more than 10% of individuals were included in the co-occurrence analyses. Lastly, we confirmed correlative results from the co-occurrence analysis by employing generalised linear mixed effect models (GLMMs) with presence of active or previous LASV infection as response variable and presence of specific MHC alleles/STs and MHC allele/ST diversity as explanatory variables, controlling for eye lens weight in milligrams (a proxy for rodent age; [46]), sex and sampling date as random effect. We used a binomial GLMM with Logit-link function, with no indication of violation of model assumptions. In cases where the co-occurrence analysis identified alleles that were correlated, GLMMs were only computed for the more frequent allele [65]. To sum up, our criteria for recognizing an allele or ST as convincingly associated with LASV were:

1. Significant association with LASV PCR and/or IgG data in the co-occurrence analyses,
2. having a higher frequency compared with correlated alleles/STs also associated with LASV in the co-occurrence analysis,
3. and being significantly linked to LASV occurrence in the complementary GLMM analysis.

All statistical analyses were performed in R using the R Studio computing environment version 2022.07.2 [66].

## 3. Results

### 3.1 MHC-I allele and supertype composition between *Mastomys* species and localities

**Fig 2:**
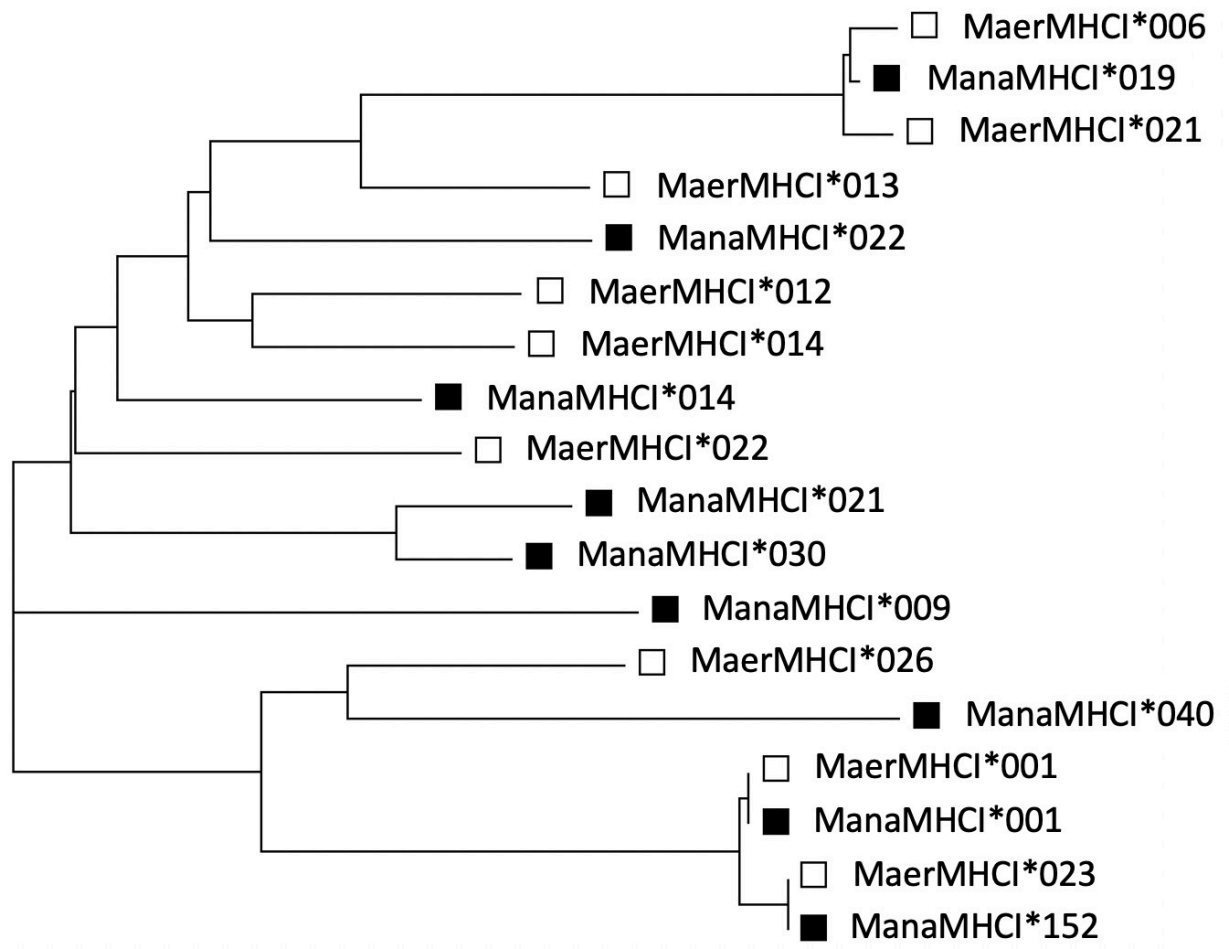
Indications of Trans-Species Polymorphism (TSP): A gene tree clade illustrating certain nucleotide sequences that are more similar between than within *M*. *natalensis* and *M*. *erythroleucus* rodents. E.g., ManaMHC-I*001 & MaerMHC-I*001. *M*. *natalensis* alleles are represented by black squares and *M*. *erythroleucus* hollow ones.

A total of 193 nucleotide alleles and 21 supertypes (STs) were obtained from 189 *M*. *natalensis* rodents, while 96 alleles and 15 STs were obtained from 67 *M*. *erythroleucus* (Table 1). Between both species, the average number of alleles per individual (28.27 ± 6.33 vs. 19.69 ± 4.97) and STs per individual (13.16 ± 2.59 vs. 8.97 ± 1.84) was significantly higher for *M*. *natalensis*, with the pattern holding true for both sampling locations. Our results indicate a difference in the number of MHC-I loci between the two species, with *M*. *natalensis* suggested to have at least 22 MHC-I loci (maximum number of alleles for an individual = 43), whereas *M*. *erythroleucus* was suggested to carry at least 15 loci (maximum number of alleles for an individual = 30). Rarefaction analysis demonstrated that the full extent of allelic diversity for *M*. *natalensis* was likely not reached, while the lower sampling effort likely captured the natural allelic diversity in *M*. *erythroleucus* (S5 Appendix). The majority of alleles were exclusive to either *M*. *natalensis* (166) or *M*. *erythroleucus* (69), though 27 sequences were shared between both species (Table 1, Fig 2; and S2 Appendix).

**Table 1:** Summarised distribution of MHC-I alleles and supertypes in two sympatric *Mastomys* species *(M*. *natalensis* and *M*. *erythroleucus)*. Rodents were trapped in two distinct sites in Nigeria: Abagboro, a non-endemic site for Lassa fever, and Ebudin, an endemic site for Lassa fever.

	*M*. *natalensis* n = 189		*M*. *erythroleucus* n = 67	
Number of individuals allocated by species and locality	Abagboro 76	Ebudin 113	Abagboro 4	Ebudin 63
Number of nucleotide alleles	27 allele sequences shared between species			
	193 {166 private}		96 {69 private}	
Mean number of nucleotide alleles per individual {range}	28.27 {9–43}		19.69 {9–30}	
Number of amino acid alleles	162		87	
Number of supertypes	21		15	
Mean number of supertypes per individual {range}	13.16 {4–19}		8.97 {5–12}	

**Fig 3:**
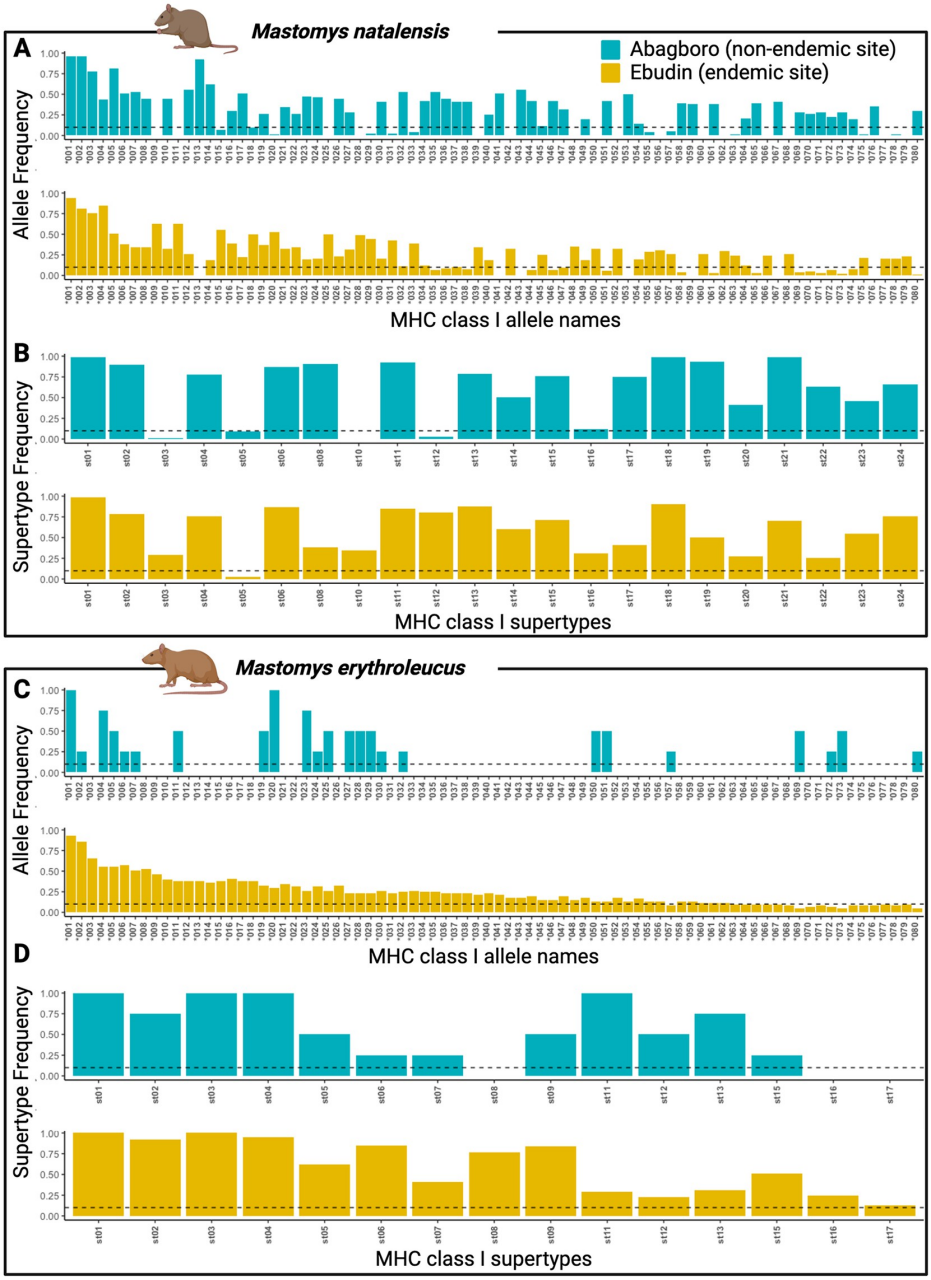
MHC-I allele and supertype distribution between *Mastomys* species and localities. Alleles *001 to *080 are shown here. Rare alleles (labelled > *080) are displayed in S2 Appendix. Colours represent the distinct localities, Abagboro (in blue) is considered non-endemic for LASV, Ebudin (in yellow) is endemic for LASV. The dotted lines represent the minimum frequency threshold of alleles and supertypes to be included in the co-occurrence analyses.

Since only four *M*. *erythroleucus* were captured in Abagboro, variation in MHC-I composition between sites could only be statistically evaluated for *M*. *natalensis*. Though 85/193 alleles and 21/22 STs were shared between sites (Table 2), dissemination of these two components of *M*. *natalensis* MHC-I composition differed significantly between Abagboro and Ebudin (Fig 3; PERMANOVA, alleles, R = 0.503, P = 0.001; STs, R = 0.2055, P = 0.001; S5 Appendix). Also, Ebudin possessed a higher proportion of private alleles (49%, 83/168) than Abagboro (22.73%, 25/110). Individual MHC allelic diversity, however, was similar between locations (Table 2 and S5 Appendix).

**Table 2:** *Mastomys natalensis* MHC-I composition between two localities in Nigeria. Abagboro is a non-endemic site for Lassa fever, and Ebudin is an endemic site for Lassa fever.

	Abagboro n = 76	Ebudin n = 113
Number of alleles	193 {85 shared}	
	110 {25 private}	168 {83 private}
Mean {range} number of alleles per individual	28.3 {9–43}	
	29.21 {12–43}	27.63 {9–39}
Number of supertypes	22 {21 shared}	
	21 {0 private}	22 {1 private}
Mean {range} number of supertypes per individual	13.21 {4–19}	
	13.47 {6–16}	12.95 {4–19}

### 3.2 MHC-I genotype and LASV infection

**Fig 4:**
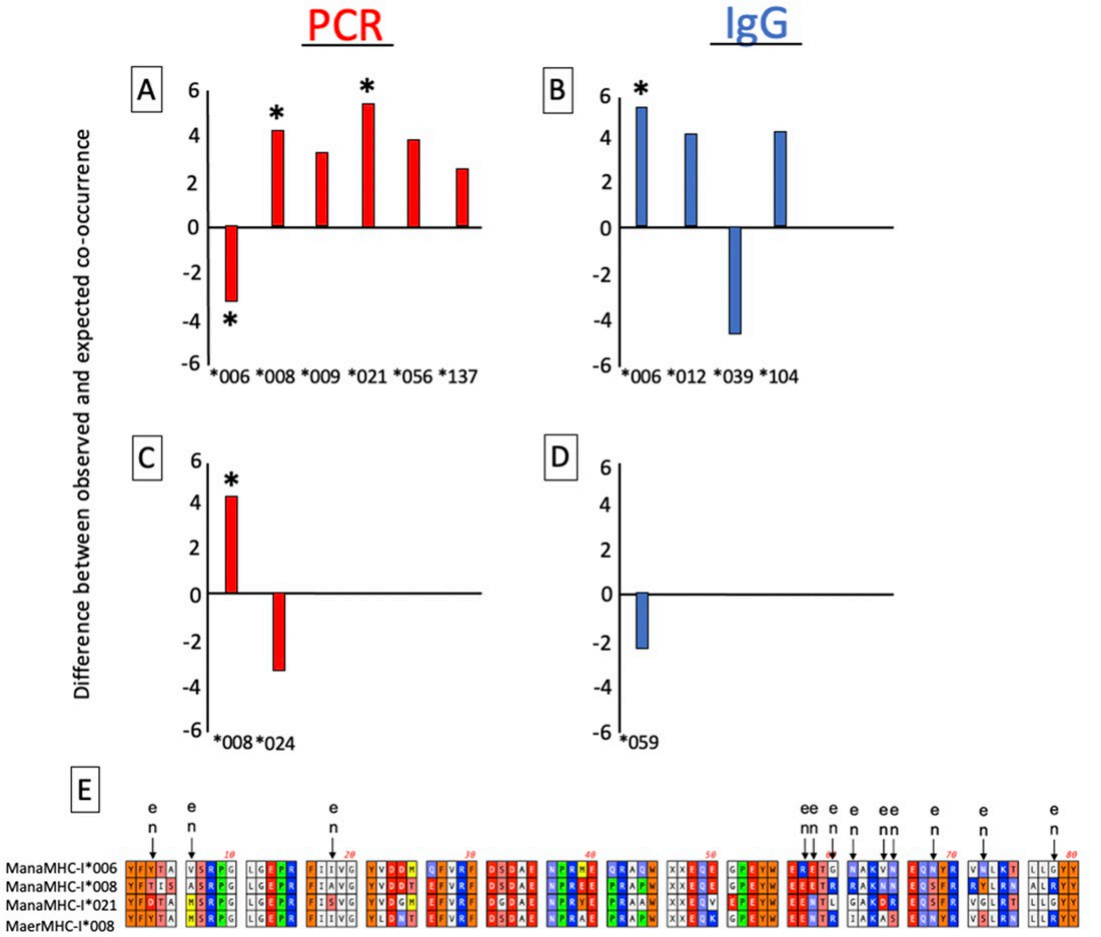
Residual co-occurrence values and sequence patterns of *Mastomys* alleles found to be significantly associated with LASV infection. A) *M*. *natalensis* alleles significantly associated with LASV PCR. B) *M*. *natalensis* alleles significantly associated with LASV IgG. C) *M*. *erythroleucus* alleles significantly associated with LASV PCR. D) *M*. *erythroleucus* alleles significantly associated with LASV IgG. Asterisks denote significant LASV-allele relationships from both the co-occurrence analyses and General Linear Mixed Effect Models (GLMMs). Mana*009, *056 and *137 were correlated with *008; Mana*012, *039 and *104 were correlated with *006; and Maer*024 was correlated with Maer*008; In this case, GLMMs were only computed for the more frequent alleles (see Methods). E) Amino acid sequences of alleles significantly associated with LASV occurrence in both the co-occurrence analyses and GLMMs, showing positively selected sites common to both *M*. *natalensis* (n) and *M*. *erythroleucus* (e).

*M*. *natalensis* and *M*. *erythroleucus* in Ebudin, the endemic zone for Lassa fever, were detected to carry active or previous LASV infections (Table 3). Conversely, both rodent species in Abagboro, the non-endemic area, were LASV-negative, except for one *M*. *natalensis* individual, which was IgG-positive. Because LASV infections were only recorded in Ebudin, our assessment of the relationship between MHC-I constitution and LASV focused on this location. Several alleles were found to be significantly associated with active and previous LASV infection in the co-occurrence analyses (Fig 4 and S6 Appendix). When MHC alleles were correlated with each other, only the allele occurring at higher frequency was investigated further in the GLMMs. Specifically, alleles ManaMHC-I*012, *039 & *104 were correlated with *006, but were less frequent (S2 and S6 Appendices). The pattern was the same for alleles ManaMHC-I*009, *056 & *137 with *008; and allele MaerMHC-I*024 with *MaerMHC-I*008. Furthermore, we only regarded LASV-allele associations from the co-occurrence analyses as reliable when they were supported by GLMM results (Fig 4 and S7 Appendix). Ultimately, alleles that satisfied these stipulations were ManaMHC-I*006, *008, *021 and MaerMHC-I*008 (Fig 4 and S6 and S7 Appendices).

**Table 3:** LASV infection profile of two *Mastomys* rodents including the number of individuals that were tested negative for LASV and those tested positive for an active LASV infection in PCRs and/or positive for LASV specific IgG antibodies.

Locality	*Mastomys* species	LASV negative	Exclusively PCR-positive	Simultaneously PCR- and IgG- positive	Exclusively IgG- positive	Total
Ebudin	*M*. *natalensis*	69	13	1	30	113
	*M*. *erythroleucus*	35	9	2	17	63
Abagboro	*M*. *natalensis*	75	0	0	1	76
	*M*. *erythroleucus*	4	0	0	0	4

Allele ManaMHC-I*006 was negatively associated with active (PCR-positive) LASV infection in *M*. *natalensis*, suggesting resistance (Fig 4A). The same allele was positively associated with previous infection (indicated by an IgG-positive result), possibly suggesting competent clearance (Fig 4B). The alleles ManaMHC-I*008 & *021 were positively associated with active infection, suggesting susceptibility (Fig 4A). In the case of *M*. *erythroleucus*, MaerMHC-I*008 was positively associated with active LASV infection (Fig 4C). Associations between STs and LASV infection status detected by co-occurrence analyses were not supported by GLMMs (S6 and S7 Appendices). Apart from the alleles above, no fixed effects or control variables (except eye lens weight, which was positively linked to previous LASV infection) were significantly associated with active or previous LASV infections (S7 Appendix).

### 3.3 Amino acid differences associated with LASV infection

Among the PSSs of the alleles linked to LASV infections (Fig 4), the three susceptibility alleles shared a Glutamic acid at position 57, whereas the resistance allele ManaMHC-I*006 presented an Arginine at the same position. A similar motif was observed at three sequence positions outside the PSS: ManaMHC-I*006 possessed residue Glutamine at positions 26 and 41, while the susceptibility alleles coded Glutamic acid and Proline, respectively. ManaMHC-I*006 also showed Lysine and the susceptibility alleles Arginine at position 74.

Bearing in mind the trans-species polymorphism hinted at in this study (Fig 2 and S2 Appendix), it is important to note that ManaMHC-I*006 & ManaMHC-I*021 are not private alleles, but have counterparts in *M*. *erythroleucus* (MaerMHC-I*020 & MaerMHC-I*062, respectively). However, the *M*. *erythroleucus* alleles were not significantly associated with LASV.

Even though we cannot directly test whether differences in allele and/or ST composition may contribute to differences in LASV prevalence between sites, it is noteworthy that ManaMHC-I*006, ManaMHC-I*008 & ManaMHC-I*021 were relatively abundant in Ebudin, where they are associated with LASV. These alleles also occurred with a high frequency in Abagboro, where LASV was not detected (Fig 3).

## 4. Discussion

Considering the importance of African multimammate mice as preeminent reservoirs of several actual and potential zoonotic pathogens that include mammarenaviruses, very little is known about the link to the immunogenetics of these animals [39]. This is key to gauge the likelihood for any such virus, such as LASV, to be transmitted between individual rodents, to emerge across populations and to spillover to humans. This study is the first to describe MHC-I sequence characteristics, immunogenetic diversity, and distribution of MHC-I alleles and supertypes between *Mastomys natalensis* and *M*. *erythroleucus* species, and between populations from the endemic and non-endemic zone for Lassa fever. Importantly, we demonstrated that specific MHC-I alleles in *Mastomys* rodents within Nigeria are linked to resistance, competent clearance, and susceptibility to LASV.

### 4.1 MHC-I constitution in *Mastomys* rodents

Information about diversity of the MHC class I in wild murid rodents is still rather limited. Wild-caught *Mus musculus* across various sites in Europe and Iran yielded 29 and 183 MHC-I alleles from exon 2 of the H2-D and H2-K loci, respectively [53], compared to the 30–40 genes found in conventional laboratory *Mus musculus*. Wild African Pygmy mice *Mus (Nannomys) setulosus* were even found to harbour several thousand MHC-I genes [67]. Our study offers a first glimpse into similar diversity of the MHC-I in *Mastomys* rodents: 193 and 96 alleles were detected in *M*. *natalensis* and *M*. *erythroleucus*, respectively. Moreover, at least 22 MHC-I loci were estimated in *M*. *natalensis*, and 15 in *M*. *erythroleucus*. Such exceptional diversity is emblematic of the MHC and commonly coupled to gene duplication [68–70]. The allelic MHC-I variability observed between *M*. *natalensis* and *M*. *erythroleucus* is not just a result of larger sample size, but more likely due to *M*. *natalensis*’ wide geographic distribution, its ecological adaptability and well-documented interaction with a spectrum of mammarenaviruses and pathogens beyond that of any other African rodent. A similarly expansive allelic MHC-I repertoire has been reported for birds and bats with a particularly wide range [71,72]. The fact that *M*. *natalensis* and *M*. *erythroleucus* share 27 alleles implies trans-species polymorphism possibly maintained by balancing selection acting from a million years ago, when both taxa are estimated to have split from their common ancestor [73]. Furthermore, 12 of 16 positively selected sites are shared by *M*. *natalensis* and *M*. *erythroleucus*, which supports the idea that both species experience similar pathogen-mediated selection.

### 4.2 Immunogenetic correlates of acute and cleared LASV infection

The majority of LASV and similar mammarenavirus infections in *Mastomys* are acute [21,44,46] and last between 2–4 weeks [45,47]. IgG antibodies commonly appear in serum 1–2 weeks after infection and are retained possibly for life [45,47]. Field data for various mammarenaviruses show that *M*. *natalensis* which simultaneously test positive by PCR and IgG assays (i.e., those that carry a chronic infection from shortly after birth, or are seroconverting) represent a minor proportion of infected individuals [21,44,46]. Our findings line up with these earlier results, as the great majority of individuals were either PCR or IgG positive, with only very few exceptions. It begs the question whether immunogenetic differences are at the root of differences in the speed of progression of virus infection as well as the swiftness of IgG responses. In *M*. *natalensis*, allele ManaMHC-I*006 co-occurred negatively with the LASV PCR- and positively with the IgG-signal. We propose that individuals bearing this allele, when challenged with LASV, clear the virus efficiently. These rodents are therefore less likely to be encountered with viremia, but more likely with mounted IgG responses. By contrast, positive associations between LASV-PCR and ManaMHC-I*008, ManaMHC-I*021 as well as MaerMHC-I*008 implied individuals were more likely to be captured with viremia, possibly due to inability to promptly clear the virus and develop protective IgG antibodies.

A mark-recapture study involving the dynamics of Morogoro virus (MORV, considered a LASV surrogate) in a natural *M*. *natalensis* population demonstrated that 15 out of 57 individuals, which tested PCR-positive with an active infection, had not turned IgG-positive upon recapture by (or, in some cases, beyond) the second week [48]. The authors hypothesize that IgG antibody development might have been delayed or was expressed in titres below the detection threshold. Our work offers a plausible explanation: the identity of MHC alleles might determine the speed at which an infection is cleared while concurrently affecting the IgG antibody response. For instance, White Leghorn chickens (*Gallus gallus domesticus*) that possessed the MHC haplotype B^13^ showed relatively high antibody titres in a lineage normally known to display low antibody response to *Brucella abortus* [74]. Likewise, minimum and maximum antibody titres to *Salmonella enteritidis*, sheep red blood cell and *Brucella abortus* are connected to MHC class I & II SNPs in chickens [75]. The association of the allele ManaMHC-I*006 with both acute infection likelihood and antibody detection points towards a mechanistic link between IgG production and MHC constitution. Future research should explore whether a link exists between MHC genotype, the timing of IgG antibody development post-infection and the amplitude of titre response.

### 4.3 Does *Mastomys* MHC-I explain LASV endemicity?

Can we explain differences in LASV prevalence between the endemic zone of Ebudin and the non-endemic area of Abagboro based on immunogenetic disparities between sites? First, *Mastomys natalensis* did not differ significantly in average number of alleles or supertypes per individual between Ebudin and Abagboro, nor was there a link between LASV and MHC diversity. Yet, there was a higher proportion of private alleles in Ebudin than Abagboro. In this sense, the endemic site contains a larger pool of rare alleles originating from diversifying selection, likely maintained by a cyclical “arms race” [30] between host immunogenetic defenses and evolving LASV lineage II variants (and/or many other pathogens) [51]. The LASV-associated alleles (ManaMHC-I*006, *008 & 021) are among the most abundant in *M*. *natalensis* (S2 Appendix). Intriguingly, however, these alleles generally registered lower percentages in Ebudin (43/113, 38%; 39/113, 35%; 37/113, 33%, respectively) than they did in Abagboro (39/76, 51%; 34/76, 45%; 26/76, 34%, respectively). Although our data cannot more than hint at possible frequency-dependent selection mechanisms in this population, changes in MHC allele frequency across space and time can be rapid in short-lived mammals [76,77] such as *Mastomys* rodents, which live to about 339 days [78]. In concert with more extensive sampling in non-endemic sites or transitional localities, extending our surveys in these populations by several more *Mastomys* generations could improve insight into how the frequency of MHC alleles fluctuates with LASV prevalence. Also, the study of T-cell lymphocytes, specially TCD8+, would be very desirable for a more complete picture of the role of immunogenetics in LASV infections.

## 5. Conclusion

We were able to identify MHC-I allele associations with LASV that conferred resistance and competent clearance and, contrariwise, susceptibility in the most important rodent reservoirs, *M*. *natalensis* and *M*. *erythroleucus*. The prevalence of mammarenaviruses in *Mastomys* populations are immediate public health concerns. Hence, expanding our surveys spatially and temporally will provide a broader view of the varied MHC-I alleles related to LASV occurrence, and their frequency distribution across *Mastomys* populations within and outside Nigeria. A better understanding of the ecology and evolution of the host, particularly with respect to interactions with humans in a populous country such as Nigeria, might help to identify spillover hotspots and predict emergence, as was done in bat-borne viruses for example [79]. Lastly, our results open the door to future research on MHC-I allele-LASV peptide combinations that advance the development of a LASV vaccine for rodents in order to regulate LASV prevalence in its most important reservoir [80,81].

## Supporting information

S1 Appendix Data obtained by illumina high-thoughput sequencing. (DOCX)

S2 Appendix MHC diversity in *Mastomys*. (XLSX)

S3 Appendix *Mastomys* MHC-I exon 2 sequence attributes. (DOCX)

S4 Appendix Allocation of MHC I alleles into supertypes. (XLSX)

S5 Appendix Statistical tests comparing MHC-I composition between *Mastomys* species and between sampling localities. (DOCX)

S6 Appendix Co-occurrence analyses of LASV and specific MHC alleles/supertypes in *Mastomys*. (DOCX)

S7 Appendix Generalized linear mixed effect models of LASV and specific MHC alleles/supertypes asssociations in *Mastomys*. (DOCX)
