# Supplementary material for: MHC-I alleles mediate clearance and antibody response to the zoonotic Lassa virus in Mastomys rodent reservoirs
Source: PLoS Negl Trop Dis. 2024 Feb 29;18(2):e0011984. doi: 10.1371/journal.pntd.0011984 (PMC10903922; doi:10.1371/journal.pntd.0011984)
Supplement: S1 Appendix — (DOCX) [file pntd.0011984.s001.docx]

**S1 Appendix: DATA OBTAINED BY ILLUMINA HIGH-THROUGHPUT SEQUENCING**

**Table S1:** MHC-I reads generated during Illumina high throughput-sequencing of *M. natalensis* and *M. erythroleucus* and the number of reads retained after bioinformatic quality control.

|  |  | ***M. natalensis*** | ***M. erythroleucus*** |
| --- | --- | --- | --- |
| Before quality control | Number of individuals originally sequenced for MHC-I | 204  {+ 28 replicates = 232 samples} | 69  {+ 8 replicates = 77 samples} |
|  | Mean number of NGS raw reads generated per sample {range} | 35,466  {2,099 – 133,179} | 39,514  {9,029 – 95,390} |
| After quality control | Mean number of reads retained per sample after quality control {range} | 17,185  {9,555 – 49,664} | 19,324  {10,981 – 52,923} |
|  | Final number of individuals retained for downstream analyses | 189 | 67 |
