# Supplementary material for: MHC-I alleles mediate clearance and antibody response to the zoonotic Lassa virus in Mastomys rodent reservoirs
Source: PLoS Negl Trop Dis. 2024 Feb 29;18(2):e0011984. doi: 10.1371/journal.pntd.0011984 (PMC10903922; doi:10.1371/journal.pntd.0011984)
Supplement: S3 Appendix — (DOCX) [file pntd.0011984.s003.docx]

**S3 APPENDIX: *MASTOMYS* MHC-I EXON 2 SEQUENCE ATTRIBUTES**

MHC alleles were 240 nucleotides in length, except for those that had indels. In *M. Natalensis*, we detected 19 alleles with deletions of 1 to 16 nucleotides and six alleles of *M. erythroleucus* had deletions ranging from 1-30 nucleotides (Table S3A). One of the *M. natalensis* alleles contained an insertion (i.e., ManaMHC-I*091, with 3 nucleotides at positions 138-140 spanning amino acid positions 46 and 47).

**Table S3A:** Occurrence of indels among *Mastomys natalensis* and *M. erythroleucus* MHC class I alleles.

| Allele | Insertion  or deletion | Nucleotide position | No of nucleotides involved | No of individuals |
| --- | --- | --- | --- | --- |
| ManaMHC-I*005 | deletion | 156-158 | 3 | 119 |
| ManaMHC-I*017 | deletion | 156-158 | 3 | 64 |
| ManaMHC-I*039 | deletion | 175 | 1 | 39 |
| ManaMHC-I*050 | deletion | 221-229 | 9 | 37 |
| ManaMHC-I*055 | deletion | 38 | 1 | 35 |
| **ManaMHC-I*091** | **insertion** | **138-140** | **3** | **22** |
| ManaMHC-I*104 | deletion | 20 | 1 | 18 |
| ManaMHC-I*105 | deletion | 16-23 | 8 | 19 |
| ManaMHC-I*109 | deletion | 207 | 1 | 19 |
| ManaMHC-I*129 | deletion | 234 | 1 | 11 |
| ManaMHC-I*131 | deletion | 44 | 1 | 11 |
| ManaMHC-I*154 | deletion | 44 | 1 | 8 |
| ManaMHC-I*163 | deletion | 30-33, 41-56 | 4, 16 | 5 |
| ManaMHC-I*169 | deletion | 77, 189-201 | 1, 13 | 2 |
| ManaMHC-I*177 | deletion | 85 | 1 | 3 |
| ManaMHC-I*182 | deletion | 69-80 | 12 | 1 |
| ManaMHC-I*183 | deletion | 69-80 | 12 | 1 |
| ManaMHC-I*187 | deletion | 69-80 | 12 | 1 |
| ManaMHC-I*194 | deletion | 69-80 | 12 | 1 |
|  |  |  |  |  |
| MaerMHC-I*015 | deletion | 153-155 | 3 | 23 |
| MaerMHC-I*028 | deletion | 20 | 1 | 16 |
| MaerMHC-I*056 | deletion | 30-43, 47-52 | 14, 6 | 8 |
| MaerMHC-I*075 | deletion | 95-100 | 6 | 5 |
| MaerMHC-I*086 | deletion | 185-214 | 30 | 4 |
| MaerMHC-I*093 | deletion | 197-209 | 13 | 3 |

Amino acid translation revealed stop codons in nine of *M. natalensis* alleles and one of *M. erythroleucus* (Table S3B).

**Table S3B:** MHC class I nucleotide alleles that contained stop-codons, and their frequency across *M. natalensis* and *M. erythroleucus* individuals, respectively.

|  | *M. natalensis* | | *M. erythroleucus* | |
| --- | --- | --- | --- | --- |
|  | Allele | No. of individuals | Allele | No. of individuals |
| Stop codons | ManaMHCI*005 | 119 | MaerMHCI*035 | 15 |
|  | ManaMHCI*087 | 20 |  |  |
|  | ManaMHCI*095 | 22 |  |  |
|  | ManaMHCI*120 | 14 |  |  |
|  | ManaMHCI*121 | 17 |  |  |
|  | ManaMHCI*124 | 16 |  |  |
|  | ManaMHCI*126 | 15 |  |  |
|  | ManaMHCI*142 | 9 |  |  |
|  | ManaMHCI*164 | 4 |  |  |

Based on the codon reading frame, we created a 240 nucleotide-long alignment that contained MHC alleles from both *Mastomys* species. Indels were included, with ManaMHC-I*091 (which had the insertion) translating into 80 amino acids and all the other alleles (including those with deletions) translating into a maximum of 78 (Figure S3). ManaMHC-I*091 occurred in 22 individuals. A total of 16 positively selected sites (PSS) were detected along the MHC-I sequence for both *M. natalensis* and *M. erythroleucus*, 12 of which were the same for both species.

**
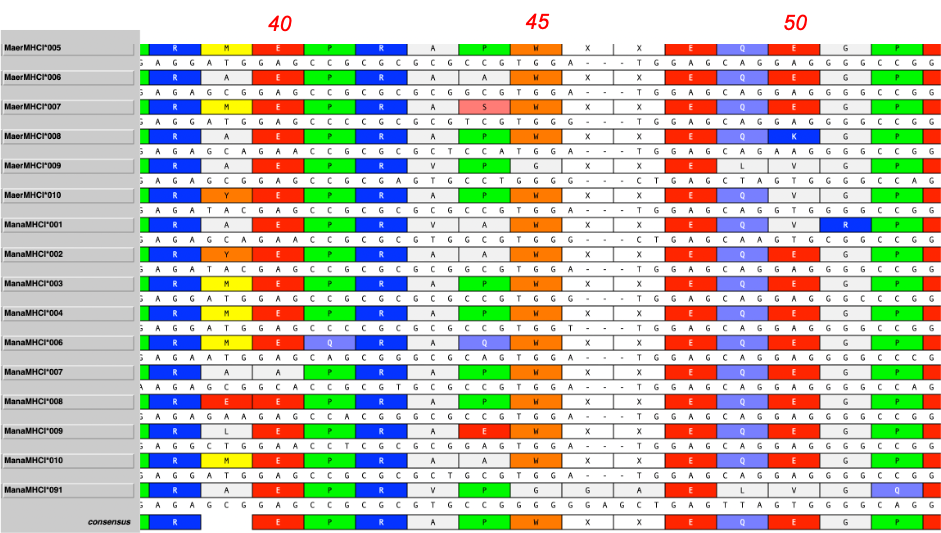
**


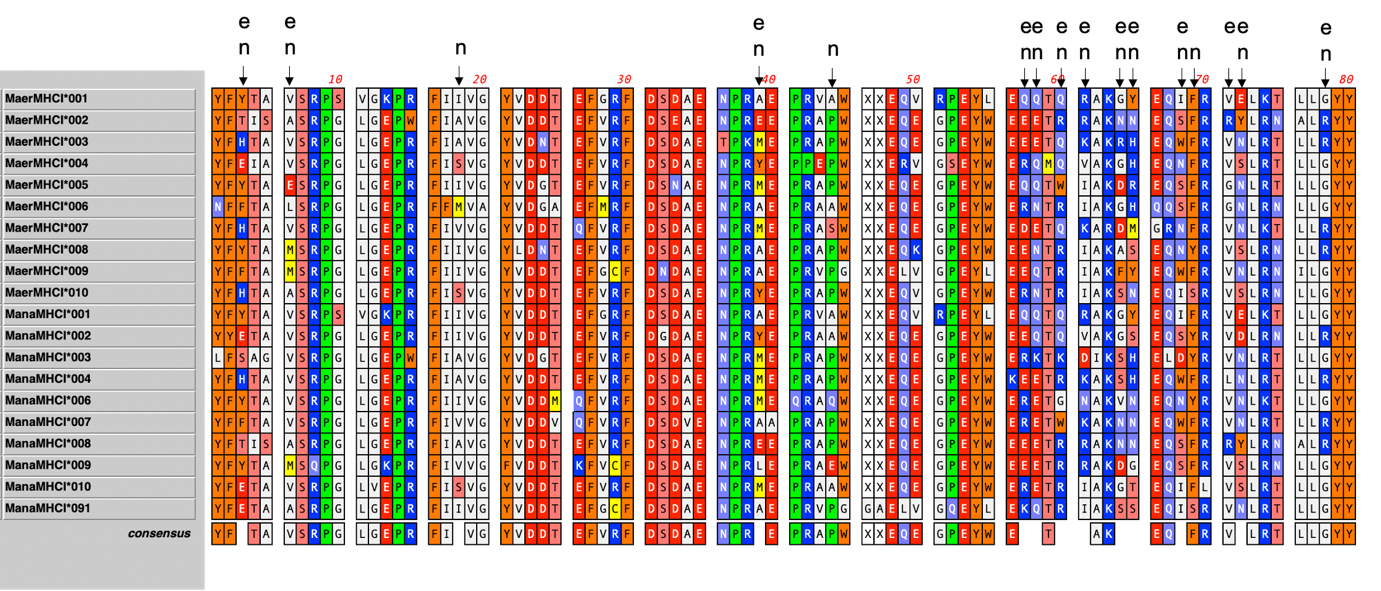


**Figure S3:** *Mastomys* MHC-I sequence alignment. The upper panel displays the insertion of three nucleotides in ManaMHC-I*091, which results into two extra amino acids (Glycine and Alanine) translated for this allele. The lower panel shows amino acid sequences of five of the most abundant alleles for *M. erythroleucus* and *M. natalensis*, respectively; in addition to the insertion-allele, ManaMHC-I*091. Arrows indicate sites along the alignment positively selected for each species (e, *M. erythroleucus*; n, *M. natalensis*).
