# Supplementary material for: MHC-I alleles mediate clearance and antibody response to the zoonotic Lassa virus in Mastomys rodent reservoirs
Source: PLoS Negl Trop Dis. 2024 Feb 29;18(2):e0011984. doi: 10.1371/journal.pntd.0011984 (PMC10903922; doi:10.1371/journal.pntd.0011984)
Supplement: S5 Appendix — (DOCX) [file pntd.0011984.s005.docx]

**S5 APPENDIX: STATISTICAL TESTS COMPARING MHC-I COMPOSITION BETWEEN *MASTOMYS* SPECIES AND BETWEEN SAMPLING LOCALITIES**

**Table S5:** Comparison of MHC class I allele and supertype diversity and composition between the rodent species *M. natalensis* and *M. erythroleucus*, trapped in two distinct localities in Nigeria (Abagboro, a non-endemic area for LASV and Ebudin, which is endemic for LASV).

| **Welch Two Sample t-test** | **t value** | **P (same mean)** |
| --- | --- | --- |
| No. of alleles per individual, *M. natalensis* (mean = 28.27) vs. *M. erythroleucus* (mean = 19.69) | -11.056 | < 2.2e-16* |
| No. of supertypes (STs) per individual, *M. natalensis* (mean = 13.16) vs. *M. erythroleucus* (mean = 8.97) | -14.075 | < 2.2e-16* |
| No. of alleles per individual, *M. natalensis* Abagboro (mean = 29.21) vs. *M. natalensis* (mean 27.63) Ebudin | 1.6838 | 0.0947 |
| No. of STs per individual, *M. natalensis* (mean = 13.47) Abagboro vs. *M. natalensis* (mean = 12.95) Ebudin | 1.3764 | 0.1842 |
| **PERMANOVA** | **R value** | **P (same mean)** |
| Allele composition, *M. natalensis* Abagboro vs. *M. natalensis* Ebudin | 0.5305 | 0.001* |
| ST composition, *M. natalensis* Abagboro vs. *M. natalensis* Ebudin | 0.2055 | 0.001* |

*statistically significant

**
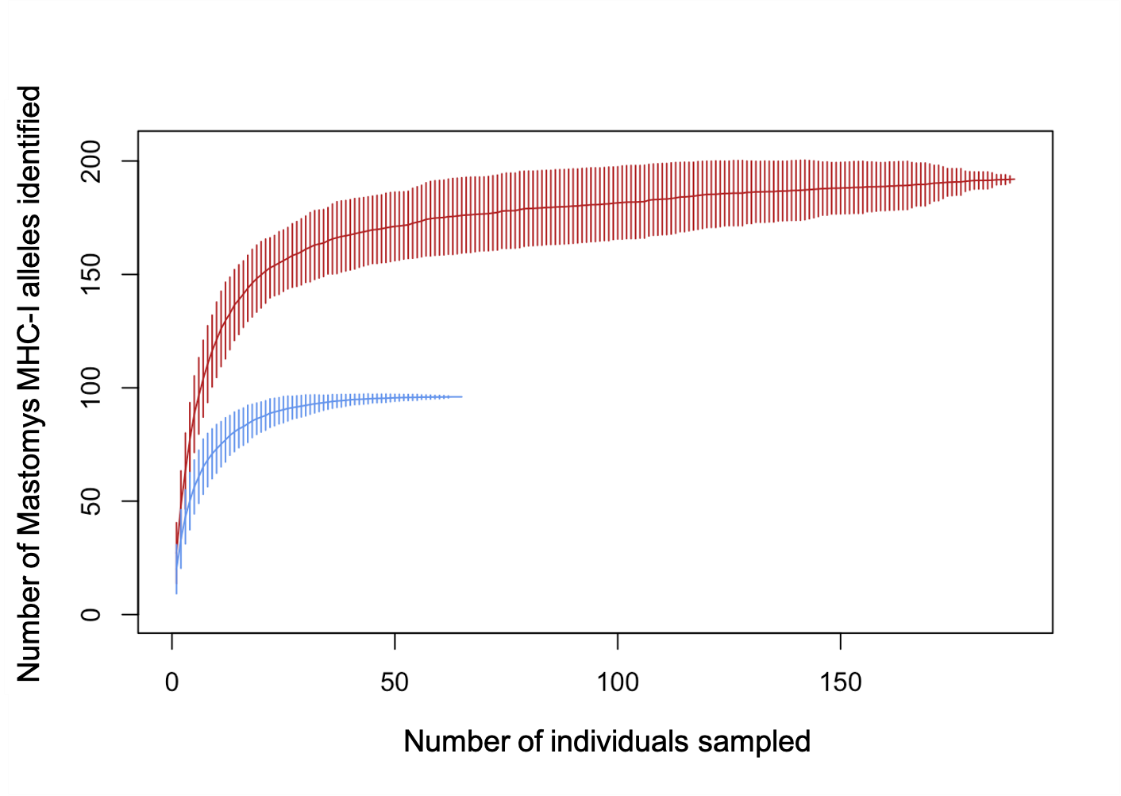
**

**Figure S5:** Rarefaction analysis. The number of detected MHC class I alleles in *Mastomys natalensis* with increasing sample size are shown in red and alleles identified in *M. erythroleucus* are shown in blue.
