# Supplementary material for: MHC-I alleles mediate clearance and antibody response to the zoonotic Lassa virus in Mastomys rodent reservoirs
Source: PLoS Negl Trop Dis. 2024 Feb 29;18(2):e0011984. doi: 10.1371/journal.pntd.0011984 (PMC10903922; doi:10.1371/journal.pntd.0011984)
Supplement: S6 Appendix — (DOCX) [file pntd.0011984.s006.docx]

**S6 APPENDIX: CO-OCCURRENCE ANALYSES OF LASV AND SPECIFIC MHC ALLELES/SUPERTYPES IN MASTOMYS.**


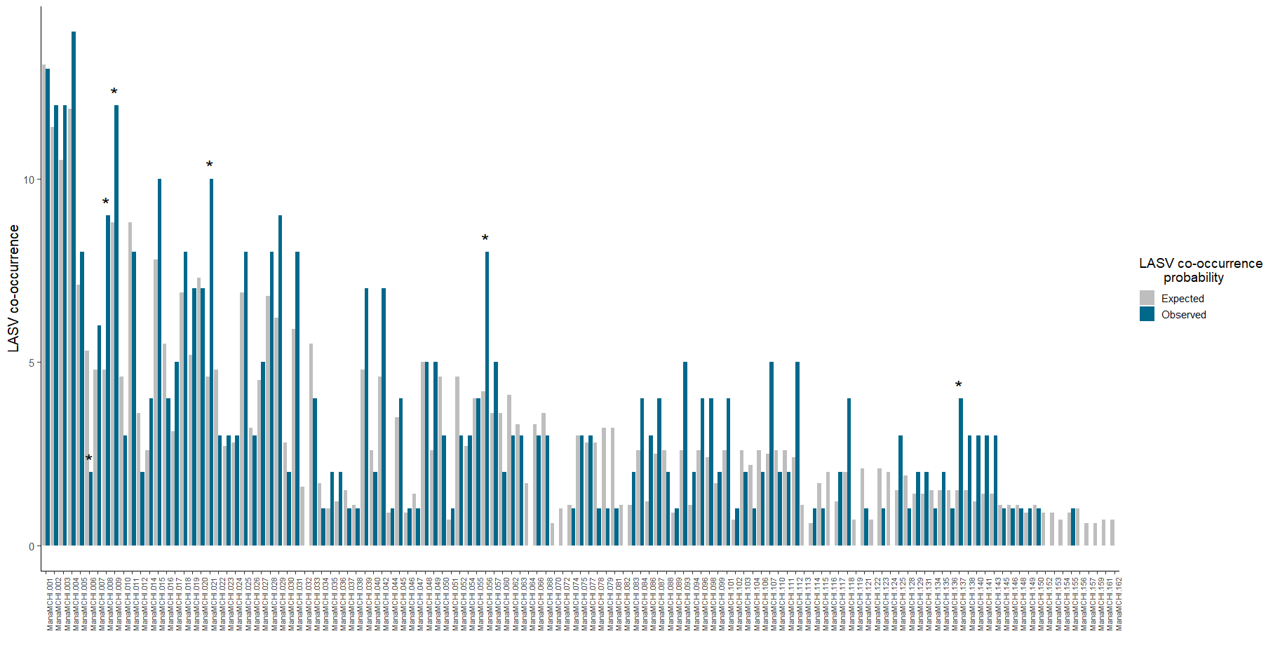
**Figure S6A:** Probability values of LASV (detected by PCR) co-occurrence with specific *Mastomys natalensis* MHC-I alleles. Asterisks indicate significant differences between expected and observed values.


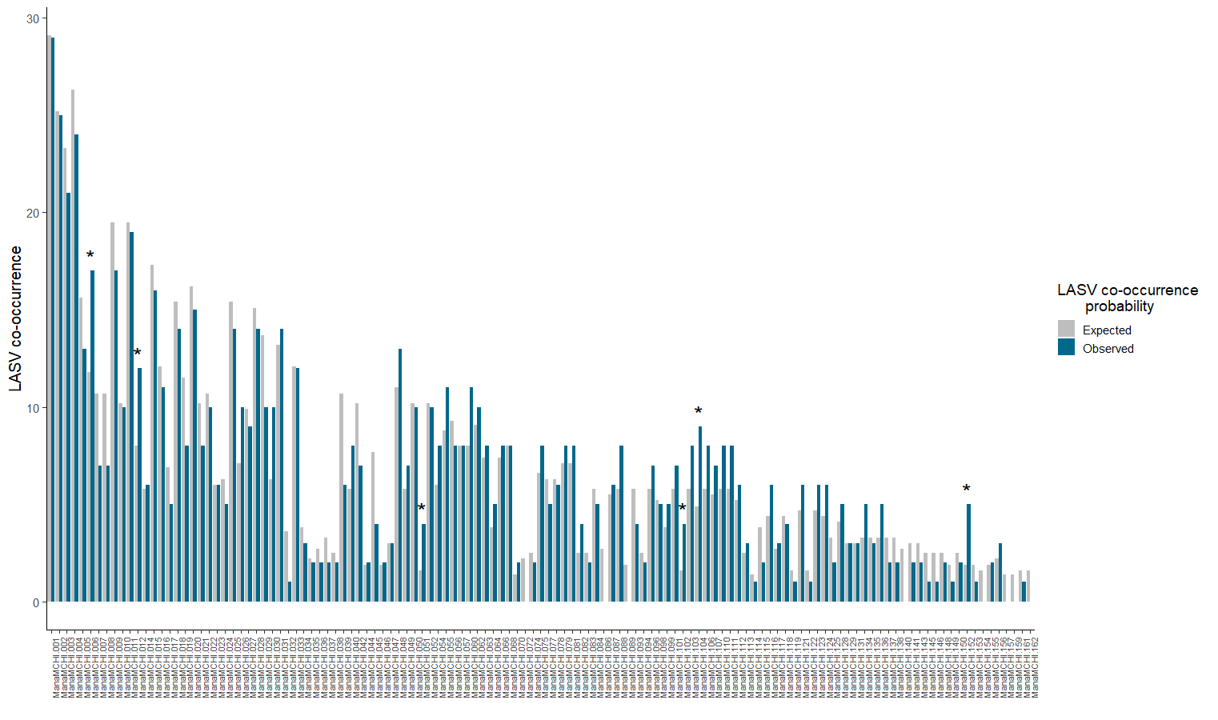


**Figure S6B:** Probability values of LASV (detected by IgG) co-occurrence with specific *Mastomys natalensis* MHC-I alleles. Asterisks indicate significant differences between expected and observed values.


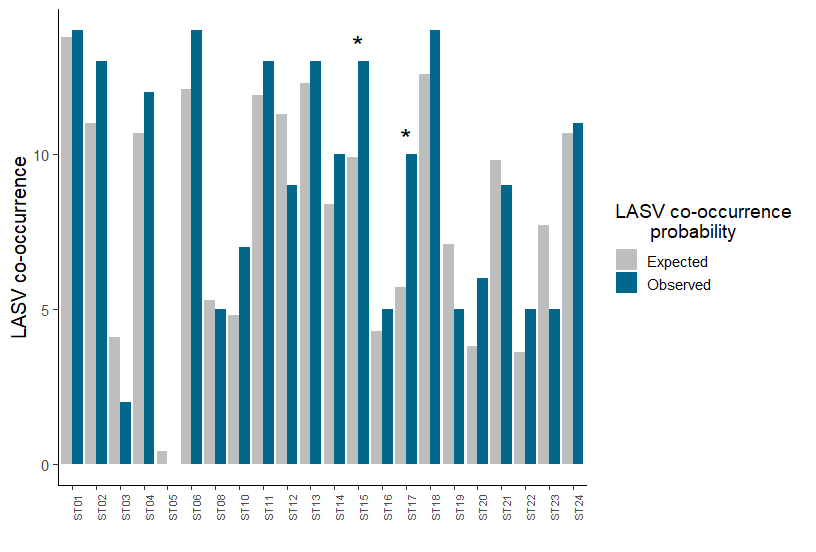


**Figure S6C:** Probability values of LASV (detected by PCR) co-occurrence with specific *Mastomys natalensis* MHC-I supertypes. Asterisks indicate significant differences between expected and observed values.


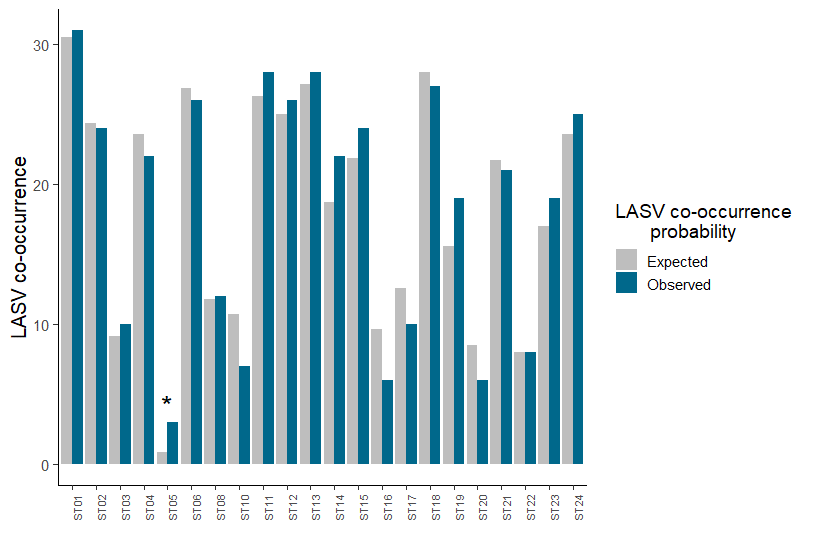


**Figure S6D:** Probability values of LASV (detected by IgG) co-occurrence with specific *Mastomys natalensis* MHC-I supertypes. Asterisks indicate significant differences between expected and observed values.


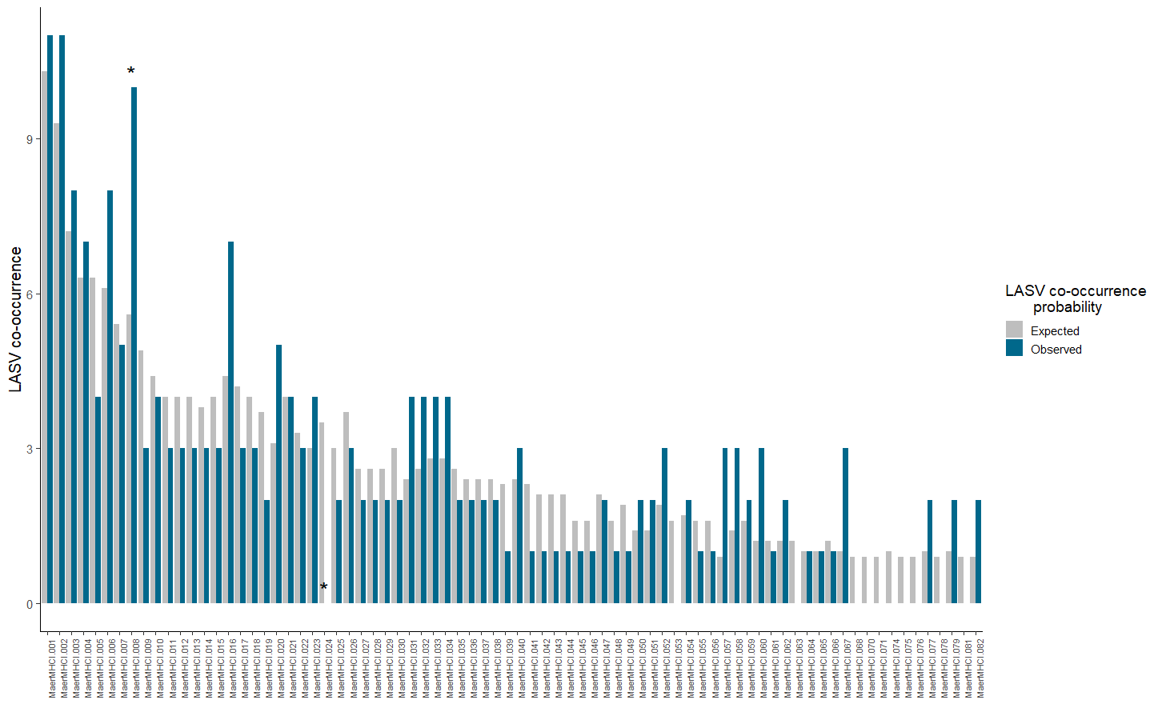


**Figure S6E:** Probability values of LASV (detected by PCR) co-occurrence with specific *Mastomys erythroleucus* MHC-I alleles. Asterisks indicate significant differences between expected and observed values.


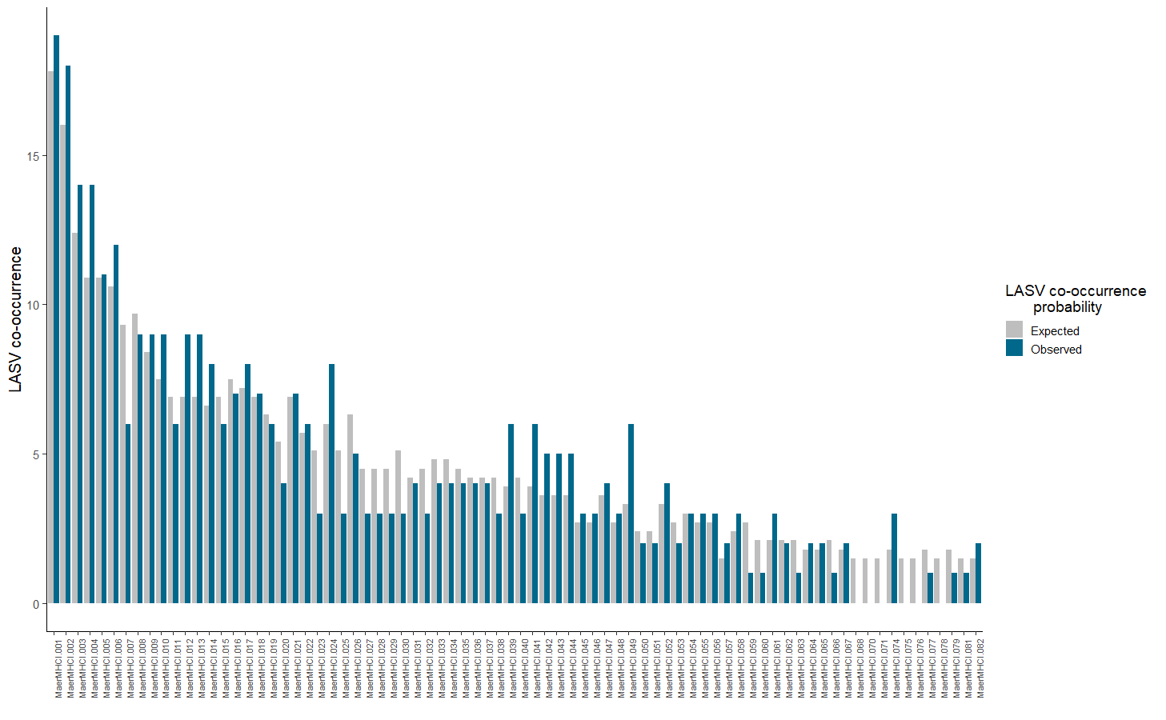


**Figure S6F:** Probability values of LASV (detected by IgG) co-occurrence with specific *Mastomys erythroleucus* MHC-I alleles. Asterisks indicate significant differences between expected and observed values.


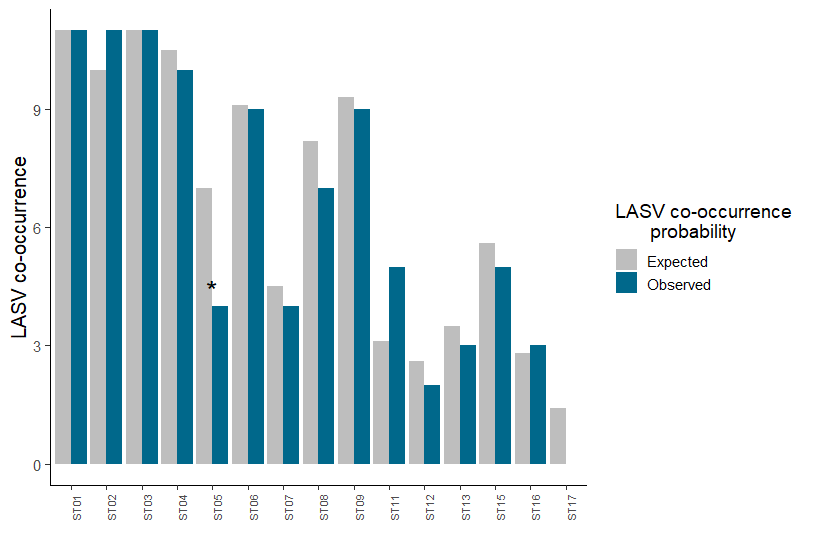


**Figure S6G:** Probability values of LASV (detected by PCR) co-occurrence with specific *Mastomys erythroleucus* MHC-I supertypes. Asterisks indicate significant differences between expected and observed values.


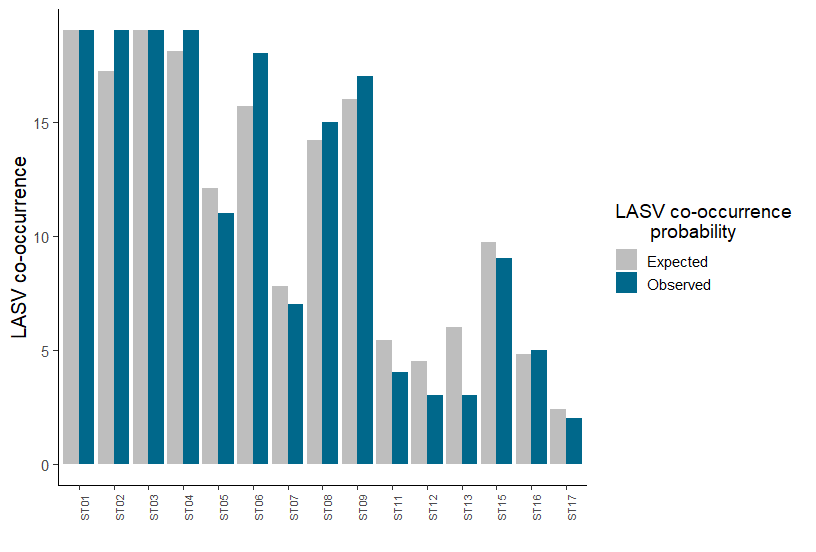


**Figure S6H:** Probability values of LASV (detected by IgG) co-occurrence with specific *Mastomys erythroleucus* MHC-I supertypes. Asterisks indicate significant differences between expected and observed values.

**Table S6A:** Correlation between alleles significantly associated with the allele ManaMHC-I*006

| **Allele comparison** | **Observed cooccurence** | **Expected cooccurence** | **Neg. associated**  **p-value** | **Pos. associated**  **p-value** |
| --- | --- | --- | --- | --- |
| ManaMHC-I*006 - ManaMHC-I*009 | 22 | 27 | 0.03542 | 0.98629 |
| ManaMHC-I*006 - ManaMHC-I*012 | 18 | 11 | 0.99949 | 0.00224 |
| ManaMHC-I*006 - ManaMHC-I*019 | 8 | 16 | 0.00108 | 0.99976 |
| ManaMHC-I*006 - ManaMHC-I*020 | 28 | 22.5 | 0.99085 | 0.02468 |
| ManaMHC-I*006 - ManaMHC-I*026 | 22 | 9.9 | 1 | 0 |
| ManaMHC-I*006 - ManaMHC-I*028 | 14 | 20.9 | 0.00609 | 0.99815 |
| ManaMHC-I*006 - ManaMHC-I*030 | 17 | 8.8 | 0.99999 | 0.00011 |
| ManaMHC-I*006 - ManaMHC-I*031 | 23 | 18.3 | 0.97984 | 0.04861 |
| ManaMHC-I*006 - ManaMHC-I*032 | 1 | 4.9 | 0.01315 | 0.99876 |
| ManaMHC-I*006 - ManaMHC-I*034 | 10 | 5.3 | 0.99871 | 0.0077 |
| ManaMHC-I*006 - ManaMHC-I*039 | 8 | 14.8 | 0.0042 | 0.99893 |
| ManaMHC-I*006 - ManaMHC-I*040 | 21 | 8 | 1 | 0 |
| ManaMHC-I*006 - ManaMHC-I*042 | 8 | 14.1 | 0.00952 | 0.99731 |
| ManaMHC-I*006 - ManaMHC-I*056 | 8 | 12.9 | 0.0287 | 0.99048 |
| ManaMHC-I*006 - ManaMHC-I*060 | 17 | 11 | 0.99776 | 0.00813 |
| ManaMHC-I*006 - ManaMHC-I*068 | 17 | 11 | 0.99776 | 0.00813 |
| ManaMHC-I*006 - ManaMHC-I*084 | 4 | 8 | 0.03769 | 0.99 |
| ManaMHC-I*006 - ManaMHC-I*088 | 21 | 8 | 1 | 0 |
| ManaMHC-I*006 - ManaMHC-I*103 | 21 | 8 | 1 | 0 |
| ManaMHC-I*006 - ManaMHC-I*104 | 15 | 6.8 | 1 | 0.00003 |
| ManaMHC-I*006 - ManaMHC-I*106 | 21 | 8 | 1 | 0 |
| ManaMHC-I*006 - ManaMHC-I*110 | 21 | 8 | 1 | 0 |
| ManaMHC-I*006 - ManaMHC-I*111 | 21 | 8 | 1 | 0 |
| ManaMHC-I*006 - ManaMHC-I*121 | 17 | 6.5 | 1 | 0 |
| ManaMHC-I*006 - ManaMHC-I*123 | 17 | 6.5 | 1 | 0 |

**Table S6B:** Correlation between alleles significantly associated with the allele ManaMHC-I*008

| **Allele comparison** | **Observed cooccurence** | **Expected cooccurence** | **Neg. associated**  **p-value** | **Pos. associated**  **p-value** |
| --- | --- | --- | --- | --- |
| ManaMHC-I*008 - ManaMHC-I*009 | 32 | 24.5 | 0.99964 | 0.00165 |
| ManaMHC-I*008 - ManaMHC-I*011 | 18 | 24.5 | 0.00722 | 0.99784 |
| ManaMHC-I*008 - ManaMHC-I*015 | 27 | 21.7 | 0.9897 | 0.02827 |
| ManaMHC-I*008 - ManaMHC-I*017 | 13 | 8.6 | 0.98889 | 0.0341 |
| ManaMHC-I*008 - ManaMHC-I*019 | 10 | 14.5 | 0.04956 | 0.9809 |
| ManaMHC-I*008 - ManaMHC-I*021 | 22 | 12.8 | 0.99998 | 0.00013 |
| ManaMHC-I*008 - ManaMHC-I*023 | 14 | 7.6 | 0.99964 | 0.00191 |
| ManaMHC-I*008 - ManaMHC-I*024 | 17 | 7.9 | 1 | 0.00002 |
| ManaMHC-I*008 - ManaMHC-I*028 | 11 | 19 | 0.00138 | 0.99967 |
| ManaMHC-I*008 - ManaMHC-I*029 | 26 | 17.3 | 0.99989 | 0.00049 |
| ManaMHC-I*008 - ManaMHC-I*030 | 12 | 7.9 | 0.9863 | 0.04184 |
| ManaMHC-I*008 - ManaMHC-I*037 | 10 | 4.1 | 0.99997 | 0.00037 |
| ManaMHC-I*008 - ManaMHC-I*039 | 9 | 13.5 | 0.04802 | 0.98204 |
| ManaMHC-I*008 - ManaMHC-I*045 | 14 | 9.7 | 0.98566 | 0.04082 |
| ManaMHC-I*008 - ManaMHC-I*054 | 14 | 7.6 | 0.99964 | 0.00191 |
| ManaMHC-I*008 - ManaMHC-I*056 | 19 | 11.7 | 0.99954 | 0.00195 |
| ManaMHC-I*008 - ManaMHC-I*078 | 3 | 7.9 | 0.01146 | 0.99781 |
| ManaMHC-I*008 - ManaMHC-I*093 | 21 | 7.2 | 1 | 0 |
| ManaMHC-I*008 - ManaMHC-I*096 | 13 | 7.2 | 0.99908 | 0.00441 |
| ManaMHC-I*008 - ManaMHC-I*101 | 13 | 7.2 | 0.99908 | 0.00441 |
| ManaMHC-I*008 - ManaMHC-I*107 | 13 | 6.9 | 0.99959 | 0.00225 |
| ManaMHC-I*008 - ManaMHC-I*112 | 13 | 6.6 | 0.99984 | 0.00105 |
| ManaMHC-I*008 - ManaMHC-I*116 | 1 | 5.5 | 0.00702 | 0.99939 |
| ManaMHC-I*008 - ManaMHC-I*118 | 13 | 5.5 | 1 | 0.00005 |
| ManaMHC-I*008 - ManaMHC-I*124 | 1 | 5.5 | 0.00702 | 0.99939 |
| ManaMHC-I*008 - ManaMHC-I*128 | 1 | 5.2 | 0.01083 | 0.99899 |
| ManaMHC-I*008 - ManaMHC-I*135 | 8 | 4.1 | 0.99685 | 0.01748 |
| ManaMHC-I*008 - ManaMHC-I*137 | 12 | 4.1 | 1 | 0 |
| ManaMHC-I*008 - ManaMHC-I*138 | 12 | 4.1 | 1 | 0 |

**Table S6C:** Correlation between alleles significantly associated with MaerMHC-I*008

| **Allele comparison** | **Observed coocccurence** | **Expected cooccurence** | **Neg. associated p-value** | **Pos. associated p-value** |
| --- | --- | --- | --- | --- |
| MaerMHC-I*008 - MaerMHC-I*016 | 25 | 13.1 | 1 | 0 |
| MaerMHC-I*008 - MaerMHC-I*024 | 6 | 10 | 0.02703 | 0.99368 |
| MaerMHC-I*008 - MaerMHC-I*034 | 14 | 7.9 | 0.99999 | 0.0002 |
| MaerMHC-I*008 - MaerMHC-I*039 | 2 | 6.8 | 0.00288 | 0.99974 |
| MaerMHC-I*008 - MaerMHC-I*041 | 2 | 6.8 | 0.00288 | 0.99974 |
| MaerMHC-I*008 - MaerMHC-I*044 | 1 | 6.3 | 0.00067 | 0.99997 |
| MaerMHC-I*008 - MaerMHC-I*052 | 11 | 5.8 | 1 | 0.00031 |
| MaerMHC-I*008 - MaerMHC-I*058 | 8 | 4.2 | 1 | 0.00357 |
| MaerMHC-I*008 - MaerMHC-I*061 | 7 | 3.7 | 1 | 0.00772 |
