# Supplementary material for: MHC-I alleles mediate clearance and antibody response to the zoonotic Lassa virus in Mastomys rodent reservoirs
Source: PLoS Negl Trop Dis. 2024 Feb 29;18(2):e0011984. doi: 10.1371/journal.pntd.0011984 (PMC10903922; doi:10.1371/journal.pntd.0011984)
Supplement: S7 Appendix — (DOCX) [file pntd.0011984.s007.docx]

**S7 Appendix: GENERALIZED LINEAR MIXED EFFECT MODELS OF LASV AND SPECIFIC MHC ALLELES/SUPERTYPES ASSOCIATIONS IN MASTOMYS.**

**Table S7A**: Model summaries of three generalised linear models testing whether LASV PCR results (positive/negative) can be explained by the *M. natalensis* MHC allele found to be associated by co-occurrence, the individual number of MHC alleles, age and sex. Significant p values are in bold and underlined.

| i) | Odds Ratio | Standard Error | p-value |
| --- | --- | --- | --- |
| ManaMHC-I*006 | 0.11 | 0.9361 | **0.021** |
| No. of alleles per individual | 1.07 | 0.05306 | 0.198 |
| Age (ELWmg) | 0.96 | 0.03872 | 0.331 |
| Sex | 0.47 | 0.69248 | 0.278 |
|  |  |  |  |
| ii) | Odds Ratio | Standard Error | p-value |
| ManaMHC-I*008 | 4.34 | 0.6710 | **0.029** |
| No. of alleles per individual | 1.00 | 0.05190 | 0.986 |
| Age (ELWmg) | 0.96 | 0.03854 | 0.309 |
| Sex | 0.62 | 0.63966 | 0.453 |
|  |  |  |  |
| iii) | Odds Ratio | Standard Error | p-value |
| ManaMHC-I*021 | 7.97 | 0.7245 | **0.004** |
| No. of alleles per individual | 0.99 | 0.0551 | 0.83 |
| Age (ELWmg) | 0.97 | 0.0397 | 0.394 |
| Sex | 0.57 | 0.6669 | 0.403 |

**Table S7B:** Model summaries of two generalised linear models testing whether LASV IgG results (positive/negative) can be explained by the *M. natalensis* MHC allele found to be associated by co-occurrence, the individual number of MHC alleles, age and sex. Significant p values are in bold and underlined.

| i) | Odds Ratio | Standard Error | p-value |
| --- | --- | --- | --- |
| ManaMHC-I*006 | 3.35 | 0.5136 | **0.019** |
| No. of alleles per individual | 0.97 | 0.0356 | 0.462 |
| Age (ELWmg) | 1.10 | 0.0294 | **0.001** |
| Sex | 0.85 | 0.4888 | 0.734 |
|  |  |  |  |
| ii) | Odds Ratio | Standard Error | p-value |
| ManaMHC-I*039 | 0.37 | 5.37e-01 | 0.067 |
| No. of alleles per individual | 1.00 | 3.32e-02 | 0.998 |
| Age (ELWmg) | 1.09 | 2.75e-02 | **0.001** |
| Sex | 0.88 | 4.58e-01 | 0.788 |

**Table S7C**: Model summaries of two generalised linear models testing whether LASV PCR results (positive/negative) can be explained by the *M. natalensis* MHC supertype (ST) found to be associated by co-occurrence, the individual number of MHC alleles, age and sex. Significant p values are in bold and underlined.

| i) | Odds Ratio | Standard Error | p-value |
| --- | --- | --- | --- |
| ST15 | ? | ? | ? |
| No. of alleles per individual | ? | ? | ? |
| Age (ELWmg) | ? | ? | ? |
| Sex | ? | ? | ? |
|  |  |  |  |
| ii) | Odds Ratio | Standard Error | p-value |
| ST17 | 3.75 | 0.7245 | 0.068 |
| No. of STs per individual | 1.06 | 0.1267 | 0.656 |
| Age (ELWmg) | 0.97 | 0.0361 | 0.347 |
| Sex | 0.77 | 0.6314 | 0.672 |

? - Model did not converge

**Table S7D:** Model summaries of two generalised linear models testing whether LASV PCR and IgG results (positive/negative) can be explained by the *M. erythroleucus* MHC allele found to be associated by co-occurrence, the individual number of MHC alleles, age and sex. Significant p values are in bold and underlined.

| i) | Odds Ratio | Standard Error | p-value |
| --- | --- | --- | --- |
| MaerMHC-I*008 | 16.79 | 1.2516 | **0.024** |
| No. of alleles per individual | 0.82 | 0.1072 | 0.067 |
| Age (ELWmg) | 1.15 | 0.1026 | 0.174 |
| Sex | 0.98 | 0.9706 | 0.984 |
|  |  |  |  |
| ii) | Odds Ratio | Standard Error | p-value |
| MaerMHC-I*059 | 0.00 | 1.25e+07 | 1.000 |
| No. of alleles per individual | 0.96 | 6.51e-02 | 0.548 |
| Age (ELWmg) | 1.12 | 4.93e-02 | **0.0183** |
| Sex | 0.40 | 6.84e-01 | 0.1844 |
